# Genetic diversity and recombination analysis of sweepoviruses from Brazil

**DOI:** 10.1186/1743-422X-9-241

**Published:** 2012-10-20

**Authors:** Leonardo C Albuquerque, Alice K Inoue-Nagata, Bruna Pinheiro, Renato O Resende, Enrique Moriones, Jesús Navas-Castillo

**Affiliations:** 1 Embrapa Vegetables, Km 09, BR060, Cx. Postal 218, Brasília, DF, CEP 70359-970, Brazil; 2 Departamento de Biologia Celular, Universidade de Brasília, CEP 70.910-970, Brasília, DF, Brazil; 3 Instituto de Hortofruticultura Subtropical y Mediterránea “La Mayora” (IHSM-UMA-CSIC), Consejo Superior de Investigaciones Científicas, Estación Experimental “La Mayora”, 29750, Algarrobo-Costa, Málaga, Spain

**Keywords:** Geminivirus, Begomovirus, Sweet potato, *Ipomoea batatas*, Convolvulaceae

## Abstract

**Background:**

Monopartite begomoviruses (genus *Begomovirus*, family *Geminiviridae*) that infect sweet potato (*Ipomoea batatas*) around the world are known as sweepoviruses. Because sweet potato plants are vegetatively propagated, the accumulation of viruses can become a major constraint for root production. Mixed infections of sweepovirus species and strains can lead to recombination, which may contribute to the generation of new recombinant sweepoviruses.

**Results:**

This study reports the full genome sequence of 34 sweepoviruses sampled from a sweet potato germplasm bank and commercial fields in Brazil. These sequences were compared with others from public nucleotide sequence databases to provide a comprehensive overview of the genetic diversity and patterns of genetic exchange in sweepoviruses isolated from Brazil, as well as to review the classification and nomenclature of sweepoviruses in accordance with the current guidelines proposed by the *Geminiviridae* Study Group of the International Committee on Taxonomy of Viruses (ICTV). Co-infections and extensive recombination events were identified in Brazilian sweepoviruses. Analysis of the recombination breakpoints detected within the sweepovirus dataset revealed that most recombination events occurred in the intergenic region (IR) and in the middle of the C1 open reading frame (ORF).

**Conclusions:**

The genetic diversity of sweepoviruses was considerably greater than previously described in Brazil. Moreover, recombination analysis revealed that a genomic exchange is responsible for the emergence of sweepovirus species and strains and provided valuable new information for understanding the diversity and evolution of sweepoviruses.

## Background

Sweet potato (*Ipomoea batatas*, family *Convolvulaceae*) is one of the most important subsistence crops in developing countries and the third most important root crop after potato (*Solanum tuberosum*) and cassava (*Manihot esculenta*) [1]. More than 30 viruses are known to infect sweet potato and in some cases cause serious diseases in this crop [2]. Many of these viruses are monopartite begomoviruses (genus *Begomovirus*, family *Geminiviridae*).

The first two sweet potato begomoviruses to be characterized at the molecular level were *Sweet potato leaf curl virus* (SPLCV) and *Sweet potato leaf curl Georgia virus* (SPLCGoV), isolated in Louisiana, USA, in 1999 [3,4]. Subsequently, begomovirus infections in sweet potato have been reported from many countries, including Peru [5], Spain [6], China [7,8], Italy [9], Uganda [10], the United States [11] and Brazil [12,13], resulting in the description of ten additional novel species [6,7,10-13]. Phylogenetically, these viruses, for which the name sweepoviruses has been proposed [14], group in a monophyletic cluster that is distinct from the two main begomovirus branches, the Old and New World groups [6,15]. In addition to sweet potato, sweepoviruses can infect other hosts such as *I. nil* or *I. setosa*[16]. The symptoms caused by sweepoviruses depend on the specific host and usually consist of leaf curling and vein yellowing, although the infection can be asymptomatic.

Begomoviruses are transmitted to dicotyledonous plants by the whitefly *Bemisia tabaci* and cause important yield losses in many crops worldwide [17-19]. They have small, circular, single-strand DNA genomes consisting of one (monopartite) or two (bipartite) components encapsidated in twinned icosahedral particles [20,21]. The viral DNA-A has one (V1) or two open reading frames (ORFs - V1 and V2, in Old World begomoviruses) in the virion sense and four ORFs (C1, C2, C3 and C4) in the complementary sense, separated by an intergenic region (IR). The DNA-A encodes the viral coat protein (CP or V1) essential for viral transmission by *B. tabaci* and a V2 protein that may potentially be involved in virus accumulation, symptom development and virus movement [22,23]. The complementary-sense strand of DNA-A encodes the replication-associated protein (Rep or C1), the transcriptional activator protein (TrAP or C2), which controls viral gene expression, the replication-enhancer protein (REN or C3), required for viral DNA replication, and C4, a suppressor of post-transcriptional gene silencing (PTGS). The DNA-B of bipartite begomoviruses encodes two proteins, the nuclear shuttle protein (NSP – BV1) and the movement protein (MP – BC1) involved in intra- and inter-cellular movement within the plant [24].

Because sweet potato plants are vegetatively propagated, accumulation of viruses may occur and results in the co-infection of multiple viral genomes in a single plant. Mixed infections of sweepovirus species and strains have been previously shown to be frequent in sweet potato [6,11,13]. This phenomenon is extremely important for virus evolution because it provides opportunities for the occurrence of natural recombination events leading to extensive viral diversity [25-27]. The importance of recombination to geminivirus evolution is well known [26,28,29], and it is probably the mechanism responsible for the genetic diversification and emergence of the most agriculturally important begomovirus species [30-32]. While generating descendants with increased fitness, recombination has also been the cause of the increased genetic diversity within the begomoviruses that consequently complicates the classification of new species.

In this report, we present a study of the genetic diversity among sweepoviruses in Brazil. Thirty-four new complete sequences were determined. Based on these new sequences and on other sequences available in public sequence databases, the classification and nomenclature of sweepoviruses were revised in accordance with the current guidelines of the *Geminiviridae* Study Group of the International Committee on Taxonomy of Viruses (ICTV). We also provide clear evidence of recombination events that may have led to the emergence of new sweepovirus strains and species.

## Results

### Sequence analysis of full-length sweepovirus genomes

The complete nucleotide sequence of 34 cloned isolates (GenBank accessions HQ393442 to HQ393472 and HQ393474 to HQ393476) corresponding to putative full-length sweepovirus genomes was determined from the sweet potato Embrapa germplasm bank (SPEGB) and from commercial field samples. All genomes (ranging from 2779 to 2843 nucleotides) had the typical organization of monopartite begomoviruses with two ORFs in the virion sense (V1 and V2) and four ORFs in the complementary sense (C1, C2, C3 and C4). All sequences contained the conserved nonanucleotide sequence 5’-TAATATT↓AC-3’ and four iterative elements (iterons, short repeated sequences important for the replication process; Additional File 1), three direct (I, II and III) and one inverted (IV), with the core consensus sequence GGWGR located around the TATA box [33]. The iteron-related domain (IRD) in the N-terminal region of the replication-associated protein (Rep IRD) was also identified [34]. Sequences were identified that contain three Rep IRDs (MATPKRFRIS, MAPPNRFKIQ and MPRAGRFNLN) that differ from those previously described by Lozano *et al.*[6] and Zhang and Ling [11] for sweepoviruses (Additional File 1).

The 34 sequences determined here were compared with the sequences of 67 sweepovirus isolates obtained from sequence databases (Table 1 and Additional File 2). Each isolate was named following the standard nomenclature for begomoviruses (Table 2; see Additional File 2 for complete isolate names). Based on the current guidelines proposed by the ICTV *Geminiviridae* Study Group [35,36], two isolates belong to the same species if the overall nucleotide identity is >89%. The isolates described in this study belong to three species, Sweet potato leaf curl virus (SPLCV), Sweet potato golden vein virus (SPGVV) (the term "associated" was eliminated from the previous name, sweet potato golden vein-associated virus, following the standard begomovirus naming recommendation) and Sweet potato leaf curl Spain virus (SPLCESV), with the percentage of nucleotide identity ranging from 92.2-98.4% within each species. Twenty-one of the isolates belong to three novel strains (the demarcation threshold for distinguishing different strains of a species is 89-94% nucleotide identity [36]) of SPLCV and SPGVV named SPLCV-Brazil (SPLCV-BR), SPLCV-Pernambuco (SPLCV-PE) and SPGVV-Rondonia (SPGVV-RO). The other isolates are variants of SPLCV-United States (SPLCV-US), SPGVV-Paraiba (SPGVV-PB) and SPLCESV. The isolates of SPGVV and SPLCESV were found only in the SPEGB, whereas SPLCV isolates were found in both the SPEGB and commercial field samples. The samples from São Paulo state were infected by both SPLCV-US and SPLCV-Sao Paulo (SPLCV-SP) isolates. However, the samples from the States of Pernambuco, Paraíba and Rio Grande do Sul were shown to be infected by SPLCV-PE (Table 2). Interestingly, we identified co-infection in six samples (#171, #184, #325, #337, #346 and #370) from the SPEGB, whereas all of the samples from commercial fields were apparently infected by a single species/strain, as suggested by the uniformity of the clones obtained from these samples (Table 2).

**Table 1:** Sweepoviruses used in this study

**Species-Strain**	**Accession no**	**Identification according to databases**	**Origin**	**Reference**	**Acronym suggested in this study**^**b**^
	**HQ393450**	**SPLCV-US[BR:PA:08]**^**a**^	**Brazil**	**This study**	**SPLCV-US[BR:PA:08]**
SPLCV-US	**HQ393451**	**SPLCV-US[BR:SE:PV:08]**	**Brazil**	**This study**	**SPLCV-US[BR:SE:PV:08]**
	**HQ393443**	**SPLCV-US[BR:BA:CA1:08]**	**Brazil**	**This study**	**SPLCV-US[BR:BA:CA1:08]**
	**HQ393446**	**SPLCV-US[BR:BA:CA2:08]**	**Brazil**	**This study**	**SPLCV-US[BR:BA:CA2:08]**
	FJ969834	SPLCV-RS2[BR:Est1]	Brazil	Paprotka et al., 2010	SPLCV-US[BR:RS:Est1:07]
	FJ969837	SPLCV-RS2[BR:Ros1]	Brazil	Paprotka et al., 2010	SPLCV-US[BR:RS:Ros1:07]
	FJ969835	SPLCV-RS2[BR:Mac1]	Brazil	Paprotka et al., 2010	SPLCV-US[BR:RS:Mac1:07]
	**HQ393471**	**SPLCV-US[BR:SP:AM1:09]**	**Brazil**	**This study**	**SPLCV-US[BR:SP:AM1:09]**
	**HQ393472**	**SPLCV-US[BR:SP:AM2:09]**	**Brazil**	**This study**	**SPLCV-US[BR:SP:AM2:09]**
	FJ969836	SPLCV-RS2[BR:Poa1]	Brazil	Paprotka et al., 2010	SPLCV-US[BR:RS:Poa1:07]
	**HQ393475**	**SPLCV-US[BR:SP:AM4:09]**	**Brazil**	**This study**	**SPLCV-US[BR:SP:AM4:09]**
	**HQ393474**	**SPLCV-US[BR:SP:AM3:09]**	**Brazil**	**This study**	**SPLCV-US[BR:SP:AM3:09]**
	HM754641	SPLCV-[Haenam1]	South Korea	GenBank	SPLCV-US[KR:Hae1:09]
	HM754637	SPLCV-[Yeojoo 507]	South Korea	GenBank	SPLCV-US[KR:Yeo507:09]
	FJ560719	SPLCKrV-[J-508]	South Korea	GenBank	SPLCV-US[KR:Yeo508:08]
	HM754639	SPLCV-[Haenam532]	South Korea	GenBank	SPLCV-US[KR:Hae532:09]
	HM754638	SPLCV-[Haenam 519–3]	South Korea	GenBank	SPLCV-US[KR:Hae519:09]
	HM754635	SPLCV-[Yeojoo 388]	South Korea	GenBank	SPLCV-US[KR:Yeo388:09]
	HM754636	SPLCV-[Nonsan 445–2]	South Korea	GenBank	SPLCV-US[KR:Non445-2:09]
	HM754634	SPLCV-[Chungju 263]	South Korea	GenBank	SPLCV-US[KR:Chu263:09]
	HM754640	SPLCV-[Haenam 618–2]	South Korea	GenBank	SPLCV-US[KR:Hae618:09]
	AF104036	SPLCV-US[US:Lou:94]	USA	Lotrakul & Valverde, 1999	SPLCV-US[US:Lou:94]
	**HQ393453**	**SPLCV-US[BR:RO:OPO:08]**	**Brazil**	**This study**	**SPLCV-US[BR:RO:OPO:08]**
	FJ176701	SPLCV-[Eastern China]	China	GenBank	SPLCV-US[CN:Jia:08]
	AB433788	SPLCV-[Japan:Kyoto:1998]	Japan	GenBank	SPLCV-US[JR:Kyo:98]
	HQ333141	SPLCV-[US:MS:WS1-4]	USA	Zhang & Ling, 2011	SPLCV-US[US:MS:WS1-4:07]
	HQ333142	SPLCV-[US:MS:WS3-8]	USA	Zhang & Ling, 2011	SPLCV-US[US:MS:WS3-8:07]
	HQ333140	SPLCV-[US:MS:4B-14]	USA	Zhang & Ling, 2011	SPLCV-US[US:MS:4b-14:07]
	HQ333139	SPLCV-[US:MS:1B-1A]	USA	Zhang & Ling, 2011	SPLCV-US[US:MS:1b-1a:07]
SPLCV-CN	EU267799	SPLCV-[RL7]	China	GenBank	SPLCV-CN[CN:Yn:RL7:07]
	FN806776	SPLCV-[Y338]	China	GenBank	SPLCV-CN[CN:Yn338:09]
	EU253456	SPLCV-[RL31]	China	GenBank	SPLCV-CN[CN:Yn:RL31:07]
SPLCV-SP	HQ393473	SPLCV-SP[BR:AlvM:09]	Brazil	Albuquerque et al., 2011	SPLCV-SP[BR:SP:AlvM:09]
	**HQ393476**	**SPLCV-SP[BR:SP:PP:09]**	**Brazil**	**This study**	**SPLCV-SP[BR:SP:PP:09]**
SPLCV-JP	AB433787	SPLCV-[Japan:Kumamoto:1998]	Japan	GenBank	SPLCV-JP[JR:Kum:98]
	AB433786	SPLCV-[Japan:Miyazaki:1996]	Japan	GenBank	SPLCV-JP[JR:Miy:96]
SPLCV-SC	HQ333138	SPLCV-[US:SC:646B-11]	USA	Zhang & Ling, 2011	SPLCV-SC[US:SC:646-B11:06]
	HQ333137	SPLCV-[US:SC:634–7]	USA	Zhang & Ling, 2011	SPLCV-SC[US:SC:634–7:06]
	HQ333135	SPLCV-[US:SC:377–23]	USA	Zhang & Ling, 2011	SPLCV-SC[US:SC:377–23:06]
	HQ333136	SPLCV-[US:SC:634–2]	USA	Zhang & Ling, 2011	SPLCV-SC[US:SC:634–2:06]
SPLCV-BR	**HQ393445**	**SPLCV-BR[BR:BA:CA:08]**	**Brazil**	**This study**	**SPLCV-BR[BR:BA:CA:08]**
	**HQ393460**	**SPLCV-BR[BR:RO:Cac:08]**	**Brazil**	**This study**	**SPLCV-BR[BR:RO:Cac:08]**
	**HQ393455**	**SPLCV-BR[BR:RO:OPO:08]**	**Brazil**	**This study**	**SPLCV-BR[BR:RO:OPO:08]**
	**HQ393449**	**SPLCV-BR[BR:SE:Ria:08]**	**Brazil**	**This study**	**SPLCV-BR[BR:SE:Ria:08]**
	**HQ393442**	**SPLCV-BR[BR:BA:Uru:08]**	**Brazil**	**This study**	**SPLCV-BR[BR:BA:Uru:08]**
SPLCV-PR	DQ644563	SPLCV-[N4]	Puerto Rico	GenBank	SPLCV-PR[PR:Me-N4:06]
	DQ644562	SPLCV-[PR80-N2]	Puerto Rico	GenBank	SPLCV-PR[PR:80-N2:06]
SPLCV-PE	**HQ393456**	**SPLCV-PE[BR:RO:PV:08]**	**Brazil**	**This study**	**SPLCV-PE[BR:RO:PV:08]**
	**HQ393464**	**SPLCV-PE[BR:RS:MP1:09]**	**Brazil**	**This study**	**SPLCV-PE[BR:RS:MP1:09]**
	**HQ393465**	**SPLCV-PE[BR:RS:MP2:09]**	**Brazil**	**This study**	**SPLCV-PE[BR:RS:MP2:09]**
	**HQ393467**	**SPLCV-PE[BR:RS:MP5:09]**	**Brazil**	**This study**	**SPLCV-PE[BR:RS:MP5:09]**
	**HQ393462**	**SPLCV-PE[BR:PE:CSF:08]**	**Brazil**	**This study**	**SPLCV-PE[BR:PE:CSF:08]**
	**HQ393463**	**SPLCV-PE[BR:PE:CSF:08]**	**Brazil**	**This study**	**SPLCV-PE[BR:PE:CSF:08]**
	**HQ393461**	**SPLCV-PE[BR:PB:PF:09]**	**Brazil**	**This study**	**SPLCV-PE[BR:PB:PF:09]**
	**HQ393466**	**SPLCV-PE[BR:RS:MP4:09]**	**Brazil**	**This study**	**SPLCV-PE[BR:RS:MP4:09]**
	**HQ393469**	**SPLCV-PE[BR:RS:MP7:09]**	**Brazil**	**This study**	**SPLCV-PE[BR:RS:MP7:09]**
	**HQ393468**	**SPLCV-PE[BR:RS:MP6:09]**	**Brazil**	**This study**	**SPLCV-PE[BR:RS:MP6:09]**
	**HQ393470**	**SPLCV-PE[BR:RS:MP3:09]**	**Brazil**	**This study**	**SPLCV-PE[BR:RS:MP3:09]**
SPLCV-Fu	FJ515898	SPLCV-[Fp-3]	China	Yang et al., 2009	SPLCV-Fu[CN:Fj:Fp3:07]
	FJ515897	SPLCV-[Fp-2]	China	Yang et al., 2009	SPLCV-Fu[CN:Fj:Fp2:07]
	FJ515896	SPLCV-[Fp-1]	China	Yang et al., 2009	SPLCV-Fu[CN:Fj:Fp1:07]
SPLCV-ES	EU856364	SPLCV-ES[ES:CI:BG12:02]	Spain	Lozano et al., 2009	SPLCV-ES[ES:CI:BG12:02]
	EF456744	SPLCV-ES[ES:CI:BG6:02]	Spain	Lozano et al., 2009	SPLCV-ES[ES:CI:BG6:02]
	EU856366	SPLCV-ES[ES:CI:BG13:02]	Spain	Lozano et al., 2009	SPLCV-ES[ES:CI:BG13:02]
SPLCV-IT	AJ586885	SPLCV-IT[IT:Sic:02]	Italy	Briddon et al., 2005	SPLCV-IT[IT:Sic:02]
SPLCLaV-BR	FJ969833	SPLCV-RS1[BR:Tav1]	Brazil	Paprotka et al., 2010	SPLCLaV-BR[BR:RS:Tav1:07]
SPLCLaV-ES	EF456746	SPLCLaV-[ES:CI:BG27:02]	Spain	Lozano et al., 2009	SPLCLaV-ES[ES:CI:BG27:02]
	EU839579	SPLCLaV-[ES:Mal:BG30:06]	Spain	Lozano et al., 2009	SPLCLaV-ES[ES:Mal:BG30:06]
SPLCBeV	FN432356	SPLCBV-[India:West Bengal:2008]	India	GenBank	SPLCBeV-[IN:Ben:08]
SPLCBRV	FJ969832	SPLCV-CE[BR:For1]	Brazil	Paprotka et al., 2010	SPLCBRV-[BR:CE:For1:07]
SPLCCaV	EF456742	SPLCCaV-[ES:CI:BG4:02]	Spain	Lozano et al., 2009	SPLCCaV-[ES:CI:BG4:02]
	EF456745	SPLCCaV-[ES:CI:BG7:02]	Spain	Lozano et al., 2009	SPLCCaV-[ES:CI:BG7:02]
	EU856365	SPLCCaV-[ES:CI:BG21:02]	Spain	Lozano et al., 2009	SPLCCaV-[ES:CI:BG21:02]
	FJ529203	SPLCCaV-[ES:CI:BG25:02]	Spain	Lozano et al., 2009	SPLCCaV-[ES:CI:BG25:02]
SPLCShV	EU309693	SPLCV	China	GenBank	SPLCShV-[CN:Sha:07]
SPLCGoV	AF326775	SPLCGV-[16]	USA	Lotrakul et al., 2003	SPLCGoV-[US:Geo:16]
MerLCuV-US	HQ333143	SPGVaV-[US:MS:1B-3]	USA	Zhang & Ling, 2011	MerLCuV-US[US:MS:1B-3:07]
MerLCuV-BR	FJ969829	SPGVaV-PA[BR:Bel1]	Brazil	Paprotka et al., 2010	MerLCuV-BR[BR:PA:Bel1:07]
MerLCuV-PR	DQ644561	MeLCV-[PR:N1]	Puerto Rico	GenBank	MeLCuV-PR[PR:N1:06]
IYVMaV	EU839576	IYVV-[ES:Mal:IG1:06]	Spain	Lozano et al., 2009	IYVMaV-[ES:Mal:IG1:06]
IYVV	EU839578	IYVV-[ES:Mal:IG5:06]	Spain	Lozano et al., 2009	IYVV-[ES:Mal:IG5:06]
	AJ132548	IYVV-[ES:98]	Spain	Banks et al., 1999	IYVV-[ES:98]
	EU839577	IYVV-[ES:Mal:IG3:06]	Spain	Lozano et al., 2009	IYVV-[ES:Mal:IG3:06]
SPLCSPV	HQ393477	SPLCSPV-[BR:AlvM:09]	Brazil	Albuquerque et al., 2011	SPLCSPV-[BR:SP:AlvM:09]
SPMV	FJ969831	SPMaV-[BR:BSB1]	Brazil	Paprotka et al., 2010	SPMV-[BR:BSB1:07]
SPLCSCV	HQ333144	SPLCSCV-[US:SC:648B-9]	USA	Zhang & Ling, 2011	SPLCSCV-[US:SC:648-B9:06]
SPLCUV	FR751068	SPLCUV-[UG:KAMP:08]	Uganda	Wasswa et al., 2011	SPLCUV-[UG:KAMP:08]
SPLCESV	EF456741	SPLCESV-[ES:CI:BG1:02]	Spain	Lozano et al., 2009	SPLCESV-[ES:CI:BG1:02]
	EF456743	SPLCESV-[ES:CI:BG5:02]	Spain	Lozano et al., 2009	SPLCESV-[ES:CI:BG5:02]
	**HQ393448**	**SPLCESV-[BR:BA:Uti:08]**	**Brazil**	**This study**	**SPLCESV-[BR:BA:Uti:08]**
	FJ151200	SPLCESV-[ES:Mal:IG2:06]	Spain	Lozano et al., 2009	SPLCESV-[ES:Mal:IG2:06]
	**HQ393458**	**SPLCESV-[BR:RO:Cac:08]**	**Brazil**	**This study**	**SPLCESV-[BR:RO:Cac:08]**
SPGVV-PB	**HQ393444**	**SPGVV-PB[BR:BA:CA:08]**	**Brazil**	**This study**	**SPGVV-PB[BR:BA:CA:08]**
	FJ969830	SPGVaV-PB1[BR:Sou1]	Brazil	Paprotka et al., 2010	SPGVV-PB[BR:PB:Sou1:07]
SPGVV-RO	**HQ393459**	**SPGVV-RO[BR:RO:Cac:08]**	**Brazil**	**This study**	**SPGVV-RO[BR:RO:Cac:08]**
	**HQ393457**	**SPGVV-RO[BR:RO:PV2:08]**	**Brazil**	**This study**	**SPGVV-RO[BR:RO:PV2:08]**
	**HQ393454**	**SPGVV-RO[BR:RO:OPO:08]**	**Brazil**	**This study**	**SPGVV-RO[BR:RO:OPO:08]**
	**HQ393447**	**SPGVV-RO[BR:BA:Uti:08]**	**Brazil**	**This study**	**SPGVV-RO[BR:BA:Uti:08]**
	**HQ393452**	**SPGVV-RO[BR:SE:PV1:08]**	**Brazil**	**This study**	**SPGVV-RO[BR:SE:PV1:08]**
SPLCCNV	DQ512731	SPLCV-[CN]	China	Luan et al., 2007	SPLCCNV-[CN:05]
	JF736657	SPLCV-B3	China	GenBank	SPLCCNV-[CN:Zhe:10]

^a^The isolates present in this study are shown in **bold**.

^b^Complete names of the viruses are shown in the Additional file 2.

**Table 2:** Origins of the 34 sweepovirus isolates used in this study

**Sample Origin**	**Sample**	**Enzyme**	**Species**	**Strain[Isolate]**	**Acronym**	**Accession No**
SPEGB^a^	#167	EcoRV	SPLCV^b^	United States[Brazil:Bahia:Cruz das Almas1:2008]	SPLCV-US[BR:BA:CA1:08]	HQ393443
SPEGB	#171	SacI		United States[Brazil:Bahia:Cruz das Almas2:2008]	SPLCV-US[BR:BA:CA2:08]	HQ393446
SPEGB	#293	EcoRV		United States[Brazil:Para:2008]	SPLCV-US[BR:PA:08]	HQ393450
SPEGB	#325	SacI		United States[Brazil:Rondonia:Porto Velho:2008]	SPLCV-US[BR:RO:PV:08]	HQ393451
SPEGB	#337	EcoRV		United States[Brazil:Rondonia:Ouro Preto do Oeste:2008]	SPLCV-US[BR:RO:OPO:08]	HQ393453
Sao Paulo	#SP12	BamHI		United States[Brazil:Sao Paulo:Alfredo Marcondes1:2009]	SPLCV-US[BR:SP:AM1:09]	HQ393471
Sao Paulo	#SP12	SacI		United States[Brazil:Sao Paulo:Alfredo Marcondes2:2009]	SPLCV-US[BR:SP:AM2:09]	HQ393472
Sao Paulo	#SP88	BamHI		United States[Brazil:Sao Paulo:Alfredo Marcondes3:2009]	SPLCV-US[BR:SP:AM3:09]	HQ393474
Sao Paulo	#SP130	BamHI		United States[Brazil:Sao Paulo:Alfredo Marcondes4:2009]	SPLCV-US[BR:SP:AM4:09]	HQ393475
Sao Paulo	#SP140	BamHI		Sao Paulo[Brazil:Sao Paulo:Presidente Prudente:2009]	SPLCV-SP[BR:SP:PP:09]	HQ393476
SPEGB	#134	SpeI		Brazil[Brazil:Bahia:Urucuca:2008]	SPLCV-BR[BR:BA:Uru:08]	HQ393442
SPEGB	#171	BamHI		Brazil[Brazil:Bahia:Cruz das Almas:2008]	SPLCV-BR[BR:BA:CA:08]	HQ393445
SPEGB	#235	BamHI		Brazil[Brazil:Sergipe:Riachao:2008]	SPLCV-BR[BR:SE:Ria:08]	HQ393449
SPEGB	#337	SacI		Brazil[Brazil:Rondonia:Ouro Preto do Oeste:2008]	SPLCV-BR[BR:RO:OPO:08]	HQ393455
SPEGB	#370	SacI		Brazil[Brazil:Rondonia:Cacoal:2008]	SPLCV-BR[BR:RO:Cac:08]	HQ393460
SPEGB	#346	EcoRV		Pernambuco[Brazil:Rondonia:Porto Velho:2008]	SPLCV-PE[BR:RO:PV:08]	HQ393456
Paraiba	#PB82	SacI		Pernambuco[Brazil:Paraiba:Pedras de Fogo:2008]	SPLCV-PE[BR:PB:PF:08]	HQ393461
Pernambuco	#PE49	BamHI		Pernambuco[Brazil:Penambuco:Camocin de São Félix1:2008]	SPLCV-PE[BR:PE:CSF1:08]	HQ393462
Pernambuco	#PE49	SacI		Pernambuco[Brazil:Pernambuco:Camocin de São Félix2:2008]	SPLCV-PE[BR:PE:CSF2:08]	HQ393463
Rio Grande do Sul	#RS9	BamHI		Pernambuco[Brazil:Rio grande do Sul:Mariana Pimentel1:2009]	SPLCV-PE[BR:RS:MP1:09]	HQ393464
Rio Grande do Sul	#RS9	SacI		Pernambuco[Brazil:Rio Grande do Sul:Mariana Pimentel2:2009]	SPLCV-PE[BR:RS:MP2:09]	HQ393465
Rio Grande do Sul	#RS24	SacI		Pernambuco[Brazil:Rio Grande do Sul:Mariana Pimentel4:2009]	SPLCV-PE[BR:RS:MP4:09]	HQ393466
Rio Grande do Sul	#RS29	BamHI		Pernambuco[Brazil:Rio Grande do Sul:Mariana Pimentel5:2009]	SPLCV-PE[BR:RS:MP5:09]	HQ393467
Rio Grande do Sul	#RS33	BamHI		Pernambuco[Brazil:Rio Grande do Sul:Mariana Pimentel6:2009]	SPLCV-PE[BR:RS:MP6:09]	HQ393468
Rio Grande do Sul	#RS52	BamHI		Pernambuco[Brazil:Rio Grande do Sul:Mariana Pimentel7:2009]	SPLCV-PE[BR:RS:MP7:09]	HQ393469
Rio Grande do Sul	#RS52	SacI		Pernambuco[Brazil:Rio Grande do Sul:Mariana Pimentel3:2009]	SPLCV-PE[BR:RS:MP3:09]	HQ393470
SPEGB	#171	BamHI	SPGVV^c^	Paraiba[Brazil:Bahia:Cruz das Almas:2008]	SPGVV-PB[BR:BA:CA:08]	HQ393444
SPEGB	#184	SacI		Rondonia[Brazil:Bahia:Utinga:2008]	SPGVV-RO[BR:BA:Uti:08]	HQ393447
SPEGB	#325	SacI		Rondonia[Brazil:Rondonia:Porto Velho1:2008]	SPGVV-RO[BR:RO:PV1:08]	HQ393452
SPEGB	#337	EcoRV		Rondonia[Brazil:Rondonia:Ouro Preto do Oeste:2008]	SPGVV-RO[BR:RO:OPO:08]	HQ393454
SPEGB	#346	EcoRV		Rondonia[Brazil:Rondonia:Porto Velho2:2008]	SPGVV-RO[BR:RO:PV2:08]	HQ393457
SPEGB	#370	BamHI		Rondonia[Brazil:Rondonia:Cacoal:2008]	SPGVV-RO[BR:RO:Cac:08]	HQ393459
SPEGB	#184	SpeI	SPLCESV^d^	[Brazil:Bahia:Utinga:2008]	SPLCESV-[BR:BA:Uti:08]	HQ393448
SPEGB	#370	BamHI		[Brazil:Rondonia:Cacoal:2008]	SPLCESV-[BR:RO:Cac:08]	HQ393458

^a^ Sweet potato Embrapa Germplasm Bank.

^b^ Sweet potato leaf curl virus.

^c^ Sweet potato golden vein virus.

^d^ Sweet potato leaf curl Spain virus.

### Taxonomic and phylogenetic analysis of sweepoviruses

Pairwise comparisons using Clustal V were performed using the sequences determined here and all full-length sweepovirus sequences available in the databases. It is worth noting that a few sweepovirus isolates were likely misclassified according to the taxonomic criteria for geminivirus classification [36] (the proposed new names are shown in Table 1 and Additional File 2). These isolates were SPLCV-Ceara[Brazil:Fortaleza1] (SPLCV-CE[BR:For1], FJ969832), SPLCV-Rio Grande do Sul1[Brazil:Tavares1] (SPLCV-RS1[BR:Tav1], FJ969833) [12], Sweet potato golden vein-associated virus-[United States:Mississipi:1b-3:07] (SPGVaV-[US:MS:1B-3], HQ333143) [11], SPGVaV-Para[Brazil:Belem1] (SPGVaV-PA[BR:Bel1], FJ969829) [12] and Ipomoea yellow vein virus-[Spain:Malaga:IG1:2006] (IYVV-[ES:Mal:IG1:06], EU839576) [6]. The genome of isolate SPLCV-CE[BR:For1] shares <89% nucleotide identity with all other begomovirus sequences (Additional File 2), and in accordance with the cut-off point of 89% identity established for species separation within the genus *Begomovirus*[35], it most likely belongs to a new species, proposed here as Sweet potato leaf curl Brazil virus (SPLCBRV). The isolate SPLCV-RS1[BR:Tav1] shares >90% identity with SPLCLaV isolates, and we therefore proposed it be classified as Sweet potato leaf curl Lanzarote virus-Brazil[BR:RS:Tav1:07] (SPLCLaV-BR[BR:RS:Tav1:07]). The IYVV-[ES:Mal:IG1:06] sequence shared <89% identity with IYVV-[Spain:1998] and all other begomovirus sequences; it is therefore suggested that it be classified as a new species named Ipomoea yellow vein Malaga virus-[ES:Mal:IG1:06] (IYVMaV-[ES:Mal:IG1:06]). In addition, based on the nucleotide sequence identities found (Additional File 2), we propose that the isolates SPGVaV-[United States:Mississippi:1B-3] (SPGVaV-[US:MS:1B-3], HQ333143), SPGVaV-[Brazil:Belém1] (SPGVaV-PA[BR:Bel1], FJ969829) and Merremia leaf curl virus-[Puerto Rico:N1] (DQ644561) be classified as strains of Merremia leaf curl virus (MerLCuV), specifically MerLCuV-US[US:MS:1B-3:07], MerLCuV-BR[BR:PA:Bel1:07] and MerLCuV-PR[PR:N1:06], respectively.

**Figure 1:**
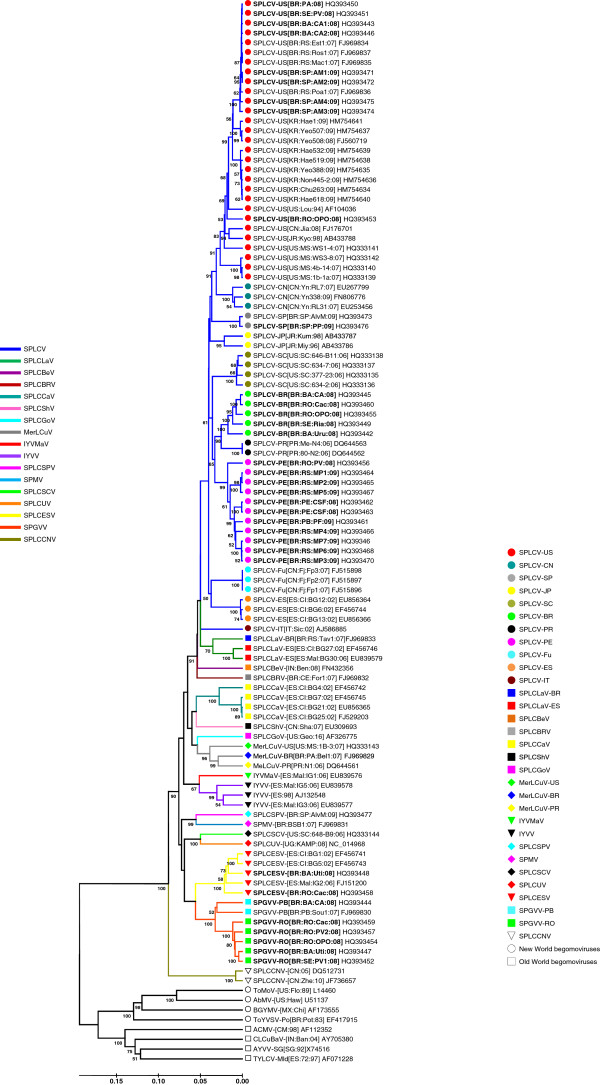
**UPGMA phylogenetic tree based on a multiple alignment of the complete sequences of the sweepoviruses described in this work (in bold) and those available in public sequence databases.** Branches were bootstrapped with 1,000 replications. Acronyms are described in Table 1. Representative sequences are included for New World (L14460, Tomato mottle virus: ToMoV-[US:Flo:89]; U51137, Abutilon mosaic virus: AbMV-[US:Haw]; AF173555, Bean golden yellow mosaic virus: BGYMV-[MX:Chi]; and EF417915, Tomato yellow vein streak virus: ToYVSV-[BR:Ba3]) and Old World begomoviruses (AF112352, African cassava mosaic virus: ACMV-[CM:98]; AY705380, Cotton leaf curl Burewala virus: CLCuBuV-[IN:Ban:04]; X74516, AYVV-SG[SG:92]; and AF071228, Tomato yellow leaf curl virus: TYLCV-Mdl[ES:72:97]). The scale bar indicates the number of substitutions per site. Bootstrap values >50% are indicated.

In the UPGMA phylogenetic tree (Figure 1), the sweepovirus sequences were consistently grouped in accordance with the proposed species/strain classification and were separated from both the Old and New World begomoviruses as was expected from the pairwise nucleotide identity analysis (Additional File 2).

### Recombination analysis

**Figure 2:**
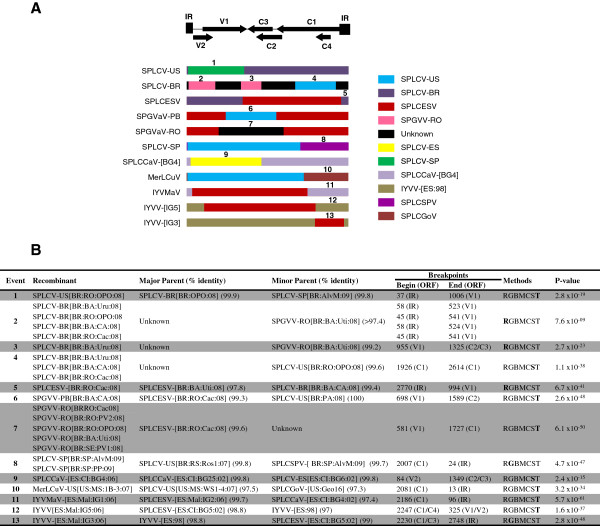
**Schematic representation of recombination events (A) and details of the recombination breakpoints (B) detected in sweepoviruses.** The genome organization of a typical sweepovirus is shown at the top of the figure. Each genome is represented by an open box, colored according to the isolate. Numbers indicate recombination events described in B. R, G, B, M, C, S and T indicate detection by RDP, GENCONV, BOOTSCAN, MAXCHI, CHIMAERA, SISCAN and 3SEQ methods, respectively, with the presented highest p-value being that determined by the method indicated in **bold** type.

**Figure 3:**
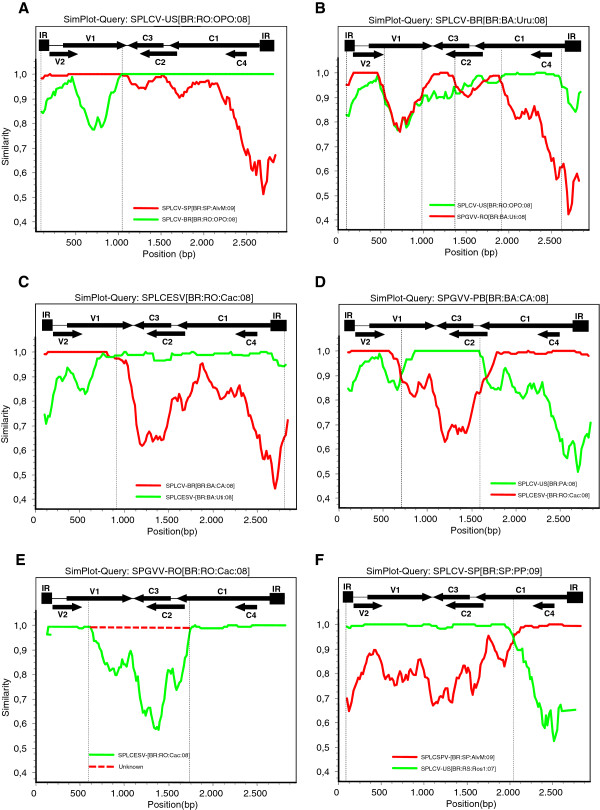
**Evidence of recombination events in Brazilian sweepoviruses (A) SPLCV-US[BR:RO:OPO:08], (B) SPLCV-BR[BR:BA:Uru:08], (C) SPLCESV-[BR:RO:Cac:08], (D) SPGVV-PB[BR:BA:CA:08], (E) SPGVV-RO[BR:RO:Cac:08] and (F) SPLCV-SP[BR:SP:PP:09].** SimPlot analyses were performed with full genome sequence alignments using the Window 200bp, Step 20bp, GapStrip on Kimura (2-parameter) method. Recombination points are shown by vertical lines.

We searched for evidence of recombination in an alignment of all 101 complete sweepovirus sequences. Different methods were used for recombination breakpoint prediction and provided strong evidence for at least 13 recombination events spread across 19 of 101 analyzed genomes (Figure 2). Remarkably, following the adopted criteria (detectable by seven different analytical methods and recombined fragments with ≥97% nucleotide identity with parental sequences), most recombination events were detected among the isolates from the SPEGB. Eight recombination patterns were detected for sequences reported in this work, while five were found in the previously published sweepovirus sequences. The recombination breakpoints were detected between the intergenic region (IR) and V1 (events 1, 2, and 5); V1 and C2/C3 (event 3); C1 and C1 (event 4), V1 and C2 (event 6), V1 and C1 (event 7); V2 and C2/C3 (event 9); C1/C4 and V1/V2 (event 12) and C1 and IR (events 8, 10, 11, and 13) (Figure 2). After analysis with the RDP3 program, the recombination events detected for the sequences reported in this study (events 1–8, Figure 2A, 2B) were tested using the SimPlot program (Figure 3). Every event identified by the RDP3 program was confirmed by Simplot. SPLCV-US[BR:RO:OPO:08] (HQ393453) appeared to be a recombinant (recombination points detected at nucleotide (nt) positions 37 and 1006) of the putative parental-like strains SPLCV-SP[BR:SP:AlvM:09] (HQ393476) and SPLCV-BR[BR:RO:OPO:08] (HQ393455) (Figure 3A). Among the isolates from the SPLCV-BR strain, SPLCV-BR[BR:BA:Uru:08] (HQ393442) contained three recombination events (event 2, breakpoints at nucleotide positions 58–523; event 3, nt positions 955–1325 and event 4, nt positions 1926–2614), three putative parental-like strains: SPGVV-RO[BR:BA:Uti:08] (HQ393447), SPLCV-US[BR:RO:OPO:08] (HQ393453) and an unknown sequence (Figure 3B). For the SPLCV-BR isolates [BR:BA:CA:08] and [BR:RO:Cac:08], two recombinant events were detected (event 2, nt positions 45–541 and event 4, nt positions 1926–2614), whereas SPLCV-BR[BR:RO:OPO:08] contained only event 2 (Figure 2B). The isolate SPLCESV-[BR:RO:Cac:08] (HQ393458) was identified as a recombinant (between nt positions 994–2770) of SPLCV-BR[BR:BA:CA:08] (HQ393445) and SPLCESV-[BR:BA:Uti:08] (HQ393448) (Figure 3C). When SPGVV-PB[BR:BA:CA:08] (HQ393444) was used as a query sequence, a recombinant breakpoint (event 6, nt positions 698–1589) with two putative parental-like viruses, SPLCESV-[BR:RO:Cac:08] and SPLCV-US[BR:PA:08] (HQ393450) (Figure 3D) was identified. In contrast, when SPGVV-RO[BR:RO:Cac:08] (HQ393459) was used as a query sequence, a recombinant breakpoint at nt positions 581–1727 (event 7) was identified with two putative parental-like viruses, SPLCESV-[BR:RO:Cac:08] and an unknown sequence (Figure 3E). The remaining four SPGVV-RO sequences contained the same recombination event observed for SPGVV-RO[BR:RO:Cac:08] (Figure 2B). Finally, when the analysis was performed for SPLCV-SP[BR:SP:PP:09] (HQ393476), two different recombination points (at nt positions 24–2007) and two putative parental strains, SPLCV-US[BR:RS:Ros1:07] (FJ969837) and SPLCSPV-[BR:SP:AlvM:09] (HQ393477), were detected (Figure 3F).

## Discussion

RCA (rolling circle amplification) has greatly facilitated the cloning of geminivirus genomes. This is especially true for sweet potato samples, as DNA extraction is difficult due to the high polysaccharide content [37]. Because RCA-based methods may start from a low amount of template DNA, sample dilution is sufficient to avoid the harmful effects of contaminating substances. In total, 34 complete sweepovirus genomes were isolated from sweet potato samples collected from a sweet potato germplasm bank (SPEGB) and commercial fields across four Brazilian states. Based on ICTV guidelines [36], the isolates belong to new strains of SPLCV [38] (strains SPLCV-BR and SPLCV-PE), and of SPGVV [12] (strain SPGVV-RO), and 13 other isolates are considered to be variants of SPLCV-US [38], SPLCV-SP [13] and SPLCESV [6].

A thorough pairwise comparison of all of the sweepovirus sequences available in the databases along with the 34 sequences reported here was performed, and it appeared that four viral isolates were not appropriately classified; hence, their classification was reviewed according to ICTV guidelines. We found that the isolates described as SPLCV-CE [12] and IYVV-[ES:IG1] [6] would be better classified as novel species, suggested here as Sweet potato leaf curl Brazil virus (SPLCBRV) and Ipomoea yellow vein Malaga virus (IYVMaV), respectively. Additionally, the SPLCV-RS1 isolate [12] may be classified as a new strain of SPLCLaV [6], and the name Sweet potato leaf curl Lanzarote virus-Brazil (SPLCLaV-BR) is proposed. Thus, it is suggested that all SPLCLaV isolates from Spain be classified as SPLCLaV-ES. Similarly, the isolates designated as SPGVaV-[US:MS:1B-3], SPGVaV-PA[BR:Bel1] and Merremia leaf curl virus-[PR:N1] (MeLCV-[PR:N1]) would be better renamed Merremia leaf curl virus-[US:MS:1B-3:07] (MerLCuV-US-[US:MS:1B-3:07]), MerLCuV-BR[BR:PA:Bel1:07] and MerLCuV-[PR:N1:06], respectively. Additionally, we suggest eliminating the term “associated” from the virus names, as symptom expression was not studied for any of the species. Finally, it was concluded that 44 of 67 sequences should be designated as strains (Table 1). This result clearly illustrates the complexity of sweepovirus taxonomy and nomenclature. Therefore, a set of modifications is suggested for updating the sweepovirus nomenclature and facilitating the interpretation of sweepovirus phylogenetic analysis (Table 1 and Figure 1).

The phylogenetic analysis demonstrated that the sweepovirus diversity found in the SPEGB samples is higher than in samples collected from commercial fields. In the SPEGB samples (n=10), three species (SPLCV, SPGVV and SPLCESV), five strains (SPLCV-US, SPLCV-BR, SPLCV-PE, SPGVV-PB and SPGVV-RO) and 11 recombinants were found, while in commercial fields (n=11), only one species (SPLCV) and three different strains (SPLCV-US, SPLCV-SP and SPLCV-PE) were observed. Moreover, co-infections were found solely in the SPEGB samples, although they have been shown to be frequent in field samples from other countries [6,11]. This could be explained by the vegetative propagation of sweet potato that favors viral accumulation in the roots, the maintenance of many sweet potato entries collected throughout Brazil and abroad in the same confined screenhouse, and the presence of whiteflies (*Bemisia tabaci*) in the germplasm bank facilities. Of the approximately 1400 entries in the SBEGB, nine are from Argentina, the USA, Paraguay, Japan and Spain, and 173 were received from the International Potato Center (CIP), Peru, as supposedly virus-free *in vitro* seedlings. The seedlings were maintained *in vivo* for at least three years, and this might have resulted in the natural spread of the begomoviruses present in some plants, thus enabling recombinations to occur. Virus-free plants or seeds will be produced to reduce the negative effect of virus infection for the breeding program.

Patterns of inter-species/strain geminivirus recombination and a number of hot- and cold-spots have been described among members of the genus *Begomovirus*[32,39-41]. We therefore analyzed the recombination breakpoints detected within the sweepovirus dataset and a number of recombination events were identified. In some cases (Figure 3A, C, D and F), the recombinants appeared to be the result of recent recombination events because a low mutation rate was observed in this region (data not shown). Most of the recombination breakpoints occur in the IR (detected between nt positions 2770 and 96) and in the middle of the C1 ORF (between nt positions 2000 and 2250) (Figure 2A). Similarly, three recombination breakpoints (events 1, 3 and 5) were identified next to the end of the V1 ORF (Figure 2A). These results are consistent with those obtained from geminivirus recombination analyses, which show that the Rep, the IR and the interface between ORFs V1 and C3 are recombination hot-spots [29,40,42,43]. Lefeuvre *et al.*[40] also described the presence of a recombination cold-spot within the V2 ORF and the third quarter of the V1 ORF of begomoviruses; here, however, we detected the occurrence of recombination breakpoints in the first half of the V1 ORF. Some sweepovirus recombination events have been previously described [6,11], and most were confirmed in our study (events 9–13, Figure 2). The detection of similar recombination patterns agrees with the recent hypothesis that the recombination sites are non-randomly distributed along the geminivirus genome [29,39,44]. The observed recombination patterns are most likely due to the existence of regions with higher biochemical and biophysical predisposition and with tolerance for recombination. The experimental generation of recombinants has shown that the IR and V1/C3 interface is a recombination hot-spot [43], and this can be explained by the fact that recombinants derived from recombination breakpoints that occur outside the genes are generally more viable than those occurring within the genes [29]. In addition to sequence homology, secondary structural features might also favor the occurrence of recombination [43]. In our case, we detected a number of recombination breakpoints in the Rep and this was also observed among other geminiviruses [26,29,45].

## Conclusions

Our study shows that the genetic diversity of sweepoviruses both in the SPEGB and commercial crops in Brazil is considerably greater than previously reported by Paprotka *et al.*[12] and highlights the importance of recombination in the evolution of these viruses. These results indicate that recombination events are apparently responsible for the emergence of sweepovirus strains and species, although alterations in host range, cell tropism, viral symptoms and pathogenicity remain to be elucidated. Recently, the generation of the first infectious clone of a sweepovirus, SPLCLaV, was described [16], which is especially important as it opens the possibility of understanding the various aspects of pathogenicity as well as its potential use in breeding virus-resistant sweet potatoes. Studies on viral diversity in particular regions provide important information enabling recommendations for viral control strategies and are essential for identifying species/strains from which to select isolates for screening tests for resistant germplasm. Finally, studies on viral diversity are necessary for comprehending how sweepovirus diversification results from propagative material exchange within the country. The ‘in vivo’ maintenance of vegetatively propagated plants in a germplasm bank has proven to be a risky strategy because it may enable or accelerate the generation of new species/strains that can spread to nature if isolation conditions are not sufficient to maintain the bank free of insect vectors.

## Methods

### Collection of leaf samples and DNA extraction

Sweet potato leaf samples showing a variety of symptoms, including vein thickening, chlorosis, curling, mottling and distortion, were collected from the sweet potato germplasm bank of Embrapa Vegetables (Brasilia-DF, Brazil) (SPEGB) and commercial fields across four Brazilian states (Table 2). Total DNA was extracted following the protocol described by Doyle and Doyle [46].

### Cloning strategy

Circular geminiviral DNA was amplified by rolling-circle amplification (RCA) using φ-29 DNA polymerase (TempliPhi kit, GE Healthcare) as described by Inoue-Nagata *et al.*[47]. RCA products were digested with a set of restriction enzymes (BamHI, EcoRI, EcoRV, HindIII, KpnI, PstI, SacI, SacII, SpeI and XbaI) to identify unique sites for cloning the full-length genomes (~2.8 kb). The restricted fragments corresponding to putative full-length monomer genomes were cloned into the vector pBluescript SK(+) (Stratagene, California, USA) and fully sequenced at Macrogen Inc. (Seoul, South Korea). One to two enzymes were finally selected for cloning the viral genomes present in each sample (Table 2).

### Genetic diversity

Pairwise nucleotide identity comparisons were calculated using Clustal V [48] (included in MegAlign DNASTAR Inc., Madison, WI, USA). As recommended by the ICTV *Geminiviridae* Study Group, viruses with nucleotide identity between full-genome sequences of <89% were considered as distinct species, while those with <94% were considered distinct strains of the same species [36].

### Phylogenetic analysis

Full genome sequences from 34 virus isolates obtained from 21 samples analyzed in this study (Table 2) and the 67 complete sweepovirus sequences available in public sequence databases ( http://www.ncbi.nlm.nih.gov/) as of October 2011 (Table 2) were aligned using Muscle [49]. The phylogenetic relationships were inferred using UPGMA with 1,000 bootstrap replicates, and the evolutionary distances were calculated using the p-distances method implemented in MEGA 5 [50].

### Recombination analysis

All sweepovirus sequences used in this study (Table 1) were aligned using Muscle with default settings [49], and the detection of potential recombinant sequences, the identification of likely parental sequences and the localization of possible recombination breakpoints were performed using RDP [51], GENCONV [26], BOOTSCAN [52], MAXICHI [53], CHIMAERA [54], SiScan [55] and 3SEQ [56] methods implemented in the RDP3 program [57]. Default settings were used throughout. Only those potential recombination events detected using all of the methods described above and involving fragments sharing ≥97% sequence identity with their parental sequences were considered. Putative recombination events were analyzed with the SimPlot program [58] using the putative recombinant sequence as a query.

## Competing interests

The authors declare that they have no competing interests.

## Authors’ contributions

LCA and BP performed the experiments. LCA, AKIN, ROR, EM and JN-C were involved in data analysis. JN-C provided overall direction and experimental design. LCA, AKIN, ROR, EM and JN-C wrote the manuscript. All authors read and approved the final manuscript.

## Supplementary Material

Additional file 1 **Iterative elements [I, II and III direct (virion-sense) and IV inverted (complementary-sense) repeats] and corresponding iteron-related domains in the 5′-terminal regions of the Rep gene (Rep IRD) of sweepoviruses.** Presumed iteron and Rep IRD sequences are colored as follows, blue for the IYVV, SPLCV-ES and SPLCV-IT group; pink for SPLCV-US[Lou:24]; and green for SPLCESV. The three different Rep IRDs present in this study are shown in **bold.** Click here for file

Additional file 2 **Sweepovirus information: Complete names and color representations of the pairwise sequence identity percentages (calculated with Clustal V, included in MegAlign-DNASTAR) of the complete genome sequences of sweepovirus isolates reported here and those available in the public sequence databases.** The isolates present in this study are shown in **bold.** Click here for file
