# Supplementary material for: Genetic diversity and recombination analysis of sweepoviruses from Brazil
Source: Virol J. 2012 Oct 20;9:241. doi: 10.1186/1743-422X-9-241 (PMC3485178; doi:10.1186/1743-422X-9-241)
Supplement: Additional file 1 — Iterative elements [I, II and III direct (virion-sense) and IV inverted (complementary-sense) repeats] and corresponding iteron-related domains in the 5′-terminal regions of the Rep gene (Rep IRD) of sweepoviruses. Presumed iteron and Rep IRD sequences are colored as follows, blue for the IYVV, SPLCV-ES and SPLCV-IT group; pink for SPLCV-US[Lou:24]; and green for SPLCESV. The three different Rep IRDs present in this study are shown in bold. [file 1743-422X-9-241-S1.pdf]

```
                                        I                          II     III
                                      --->                       --->    --->
SPLCV-BR[BR:BA:CA:08]         GCCATTTGGTGACACTCAGACTTTCAAAT-GAATTGGTG--ACTGGTGAC 2716
SPLCV-BR[BR:RO:Cac:08]        GCCATTTGGTGACACTCAGACTTTCAAAT-GAATTGGTG--ACTGGTGAC 2716
SPLCV-BR[BR:SE:Ria:08]        GCCATTTGGTGACACTCAGACTTTCAAAT-GAATTGGTG--ACTGGTGAC 2716
SPLCV-BR[BR:RO:OPO:08]        GCCATTTGGTGACACTCAGACTTTCAAAT-GAATTGGTG--ACTGGTGAC 2716
SPLCV-BR[BR:BA:Uru:08]        GCCATTTGGAGACAACCACTAGTTCAAAT-GAATTGGAG--ACTGGAGAC 2715
SPLCV-PE[BR:PE:CSF1:08]       GCCATTTGGAGACAATCAATAATTCAAAT-GAATTGGAG--ACTGGAGAC 2716
SPLCV-PE[BR:PE:CSF2:08]       GCCATTTGGAGACAATCAATAATTCAAAT-GAATTGGAG--ACTGGAGAC 2716
SPLCV-PE[BR:RS:MP7:09]        GCCATTTGGAGACAATCACTAGTTCAAAT-GAATTGGAG--ACTGGAGAC 2715
SPLCV-PE[BR:RS:MP3:09]        GCCATTTGGAGACAATCACTAGTTCAAAT-GAATTGGAG--ACTGGAGAC 2715
SPLCV-PE[BR:RS:MP6:09]        GCCATTTGGAGACAATCACTAGTTCAAAT-GAATTGGAG--ACTGGAGAC 2715
SPLCV-PE[BR:RS:MP4:09]        GCCATTTGGAGACAATCACTAGTTCAAAT-GAATTGGAG--ACTGGAGAC 2716
SPLCV-PE[BR:PB:PF:08]         GCCATTTGGAGACAATCATAATTTCAAAT-GAATTGGAG--ACTGGAGAC 2717
SPLCV-PE[BR:RS:MP1:09]        GCCATTTGGTGACACTCATACTTTCAAAT-GAATTGGTG--ACTGGTGAC 2717
SPLCV-PE[BR:RS:MP2:09]        GCCATTTGGTGACACTCATACTTTCAAAT-GAATTGGTG--ACTGGTGAC 2717
SPLCV-PE[BR:RS:MP5:09]        GCCATTTGGTGACACTGATACTTTCAAAT-GAATTGGTG--ACTGGTGAC 2716
SPLCV-PE[BR:RO:PV:08]         GCCATTTGGTGACACTCAGACTTTCAAAATGAATTGGTG--ACTGGTGAC 2717
SPLCV-US[BR:RO:OPO:08]        GCCATTTGGTGACACTCAGACTTTCAAAT-GAATTGGTG--ACTGGTGAC 2717
SPLCV-US[BR:BA:CA1:08]        GCCATTTGGAGACACGCATAAGTTCAAAT-GAATTGGAG--ACTGGAGAC 2717
SPLCV-US[BR:PA:08]            GCCATTTGGAGACACGCATAAGTTCAAAT-GAATTGGAG--ACTGGAGAC 2717
SPLCV-US[BR:BA:CA2:08]        GCCATTTGGAGACACGCATAAGTTCAAAT-GAATTGGAG--ACTGGAGAC 2717
SPLCV-US[BR:RO:PV:08]         GCCATTTGGAGACACGCATAAGTTCAAAT-GAATTGGAG--ACTGGAGAC 2717
SPLCV-US[BR:SP:AM1:09]        GCCATTTGGAGACACGCATAAGTTCAAAT-GAATTGGAG--ACTGGAGAC 2717
SPLCV-US[BR:SP:AM2:09]        GCCATTTGGAGACACGCATAAGTTCAAAT-GAATTGGAG--ACTGGAGAC 2717
SPLCV-US[BR:SP:AM3:09]        GCCATTTGGAGACACGCATAAGTTCAAAT-GAATTGGAG--ACTGGAGAC 2717
SPLCV-US[BR:SP:AM4:09]        GCCATTTGGAGACACGCATAAGTTCAAAT-GAATTGGAG--ACTGGAGAC 2715
SPLCV-SP[BR:SP:PP:09]         GTCAAATGGTGG-ACACAAAAACTCTATATGAATTGGTGGAACGGTGGAC 2733
SPGVV-RO[BR:BA:Uti:08]        ATCAATGGGTGG-ACACACAAACTCTATATGAATTGGTGGAAGGGTGGGC 2730
SPGVV-RO[BR:RO:PV1:08]        AGCTATGGGTGG-ACACACAAACTCTATATGAATTGGTGGAAGGGTGGAC 2731
SPGVV-RO[BR:RO:Cac:08]        AGCAATGGGTGG-ACACACAAACTCTATATGAATTGGTGGAAGGGTGGAC 2730
SPGVV-RO[BR:RO:OPO:08]        AGCAATGGGTGG-ACACACAAACTCTATATGAATTGGTGGAAGGGTGGAC 2730
SPGVV-RO[BR:RO:PV2:08]        AGCAATGGGTGG-ACACACAAACTCTATATGAATTGGTGGAAGGGTGGAC 2730
SPLCESV-[BR:BA:Uti:08]        ATCAATGGGTGG-ACACACAAACTCTATATGAATTGGTGGAAGGGTGGGC 2675
SPLCESV-[BR:RO:Cac:08]        AGCAATGGGTGG-ACACACAAACTCTATATGAATTGGTGGAAGGGTGGAC 2714
SPGVV-PB[BR:BA:CA:08]         AGCAATGGGTGG-ACACACAAACTCTATATGAATTGGTGGAAGGGTGGAC 2730
                              .*::: **:*. *.  * :.  **:*:: *******:*  *  *  *.*

                                          IV                                          Rep IRD
                                         <---
SPLCV-BR[BR:BA:CA:08]         AATTTATACTATGTCACCAAATGGCATTTTGGTAATTTAGAAGATCCTTT 2766 MAPPKRFRIS
SPLCV-BR[BR:RO:Cac:08]        AATTTATACTATGTCACCAAATGGCATTTTGGTAATTTAGAAGATCCTTT 2766 MAPPKRFRIS
SPLCV-BR[BR:SE:Ria:08]        AATTTATACTATGTCACCAAATGGCATTTTGGTAATTTAGAAGATCCTTT 2766 MATPKRFRIS
SPLCV-BR[BR:RO:OPO:08]        AATTTATACTATGTCACCAAATGGCATTTTGGTAATTTAGAAGATCCTTT 2766 MAPPKRFRIS
```

```
SPLCV-BR[BR:BA:Uru:08]      AATATATAGTATGTCTCCAAATGGCATTTTGGTAATTTAGAAGATCCTTT 2765 MAPPKRFKIQ
SPLCV-PE[BR:PE:CSF:09]      AATATATAGTATGTCTCCAAATGGCATTTTGGTAATTAAGAAGATCCTTT 2766 MAPPKRFKIQ
SPLCV-PE[BR:PE:CSF:09]      AATATATAGTATGTCTCCAAATGGCATTTTGGTAATTAAGAAGATCCTTT 2766 MAPPKRFKIQ
SPLCV-PE[BR:RS:MP7:09]      AATATATAGTATGTCTCCAAATGGCATTTTGGTAATTTAGAAGATCCTTT 2765 MAPPKRFKIQ
SPLCV-PE[BR:RS:MP3:09]      AATATATAGTATGTCTCCAAATGGCATTTTGGTAATTTAGAAGATCCTTT 2765 MAPPKRFKIQ
SPLCV-PE[BR:RS:MP6:09]      AATATATAGTATGTCTCCAAATGGCATTTTGGTAATTTAGAAGATCCTTT 2765 MAPPKRFKIQ
SPLCV-PE[BR:RS:MP4:09]      AATATATAATATGTCTCCAAATGGCATTTTGGTAATTTAGAAGATCCTTT 2766 MAPPKRFKIQ
SPLCV-PE[BR:RS:PF:09]       AATATATAGTATGTCTCCAAATGGCATTTTGGTAATTTAGAAGATCCTTT 2767 MAPPKRFKIQ
SPLCV-PE[BR:RS:MP1:09]      AATTTATACTATGTCACCAAATGGCATTTTGGTAATTTAGAAGATCCTTT 2767 MAPPKRFRIS
SPLCV-PE[BR:RS:MP2:09]      AATTTATACTATGTCACCAAATGGCATTTTGGTAATTTAGAAGATCCTTT 2767 MAPPKRFRIS
SPLCV-PE[BR:RS:MP5:09]      AATTTATACTATGTCACCAAATGGCATTTTGGTAATTTAGAAGATCCTTT 2766 MAPPKRFRIS
SPLCV-PE[BR:RO:PV:08]       AATTTATACTATGTCACCAAATGGCATTTTGGTAATTTAGAAGATCCTTT 2767 MAPPKRFRIS
SPLCV-US[BR:RO:OPO:08]      AATTTATACTATGTCACCAAATGGCATTTTGGTAATTTAGAAGATCCTTT 2767 MAPPKRFRIS
SPLCV-US[BR:BA:CA1:08]      AATATATAGTATGTCTCCAAATGGCATTCTGGTAATTTAGAAGATCCTTT 2767 MAPPKRFKIQ
SPLCV-US[BR:PA:08]          AATATATAGTATGTCTCCAAATGGCATTCTGGTAATTTAGAAGATCCTTT 2767 MAPPKRFKIQ
SPLCV-US[BR:BA:CA2:08]      AATATATAGTATGTCTCCAAATGGCATTCTGGTAATTTAGAAGATCCTTT 2767 MAPPNRFKIQ
SPLCV-US[BR:RO:PV:08]       AATATATAGTATGTCTCCAAATGGCATTCTTGTAATTTAGAAGATCCTTT 2767 MAPPKRFKIQ
SPLCV-US[BR:SP:AM1:09]      AATATATAGTATGTCTCCAAATGGCATTCTGGTAATTTAGAAGATCCTTT 2767 MAPPKRFKIQ
SPLCV-US[BR:SP:AM2:09]      AATATATAGTATGTCTCCAAATGGCATTCTGGTAATTTAGAAGATCCTTT 2767 MAPPKRFKIQ
SPLCV-US[BR:SP:AM3:09]      AATATATAGTATGTCTCCAAATGGCATTCTGGTAATTTAGAAGATCCTTT 2767 MAPPKRFKIQ
SPLCV-US[BR:SP:AM4:09]      AATATATAGTATGTCTCCAAATGGCATTCTGGTAATTTAGAAGATCCTTT 2765 MAPPKRFKIQ
SPLCV-SP[BR:SP:PP:09]       AATTTATATGTGTCCACCAAATGGCAAATTGGTAATTTAG--GTAACTTT 2781 MPRAGRFNIK
SPGVV-RO[BR:BA:Uti:08]      AATTTATATGTGTCCACCTAATGGCATTTTGGTAATTAG---------AA 2771 MPRAGRFNIN
SPGVV-RO[BR:RO:PV1:08]      AATTTATATGTGTCCACCTAATGGCATTTTGGTAATTAG---------AA 2772 MPRAGRFNIN
SPGVV-RO[BR:RO:Cac:08]      AATATATATGTGTCCACCTAATGGCATTTTGGTAATTAG---------AA 2771 MPRAGRFNIN
SPGVV-RO[BR:RO:OPO:08]      AATTTATATGTGTCCACCTAATGGCATTTTGGTAATTAG---------AA 2771 MPRAGRFNIN
SPGVV-RO[BR:RO:PV2:08]      AATTTATATGTGTCCACCTAATGGCATTTTGGTAATTTAGAAGATCCTTT 2780 MPRAGRFNLN
SPLCESV-[BR:BA:Uti:08]      AATTTATATGTGCCCACCTAATGGCATTTTGGTAATTAG---------AA 2716 MPRAGRFNIN
SPLCESV-[BR:RO:Cac:08]      AATATATATGTGTCCACCTAATGGCATTTTGGTAATTAG---------AA 2755 MPRAGRFNIN
SPGVV-PB[BR:CA:08]          AATTTATATGTGTCCACCTAATGGCATTTTGGTAATTAG---------AA 2771 MPRAGRFNIN
                            ***:****  :   *:**:*******:: * ******:.
```
