# Supplementary material for: Genetic diversity and recombination analysis of sweepoviruses from Brazil
Source: Virol J. 2012 Oct 20;9:241. doi: 10.1186/1743-422X-9-241 (PMC3485178; doi:10.1186/1743-422X-9-241)

Species	strain[isolate]	Acronym
Sweet potato leaf curl virus	**United States[Brazil:Para:2008]**	**SPLCV-US[BR:PA:08]**
	**United States[Brazil:Rondonia:Porto Velho:2008]**	**SPLCV-US[BR:SE:PV:08]**
	**United States[Brazil:Bahia:Cruz das Almas1:2008]**	**SPLCV-US[BR:BA:CA1:08]**
	**United States[Brazil:Bahia:Cruz das Almas2:2008]**	**SPLCV-US[BR:BA:CA2:08]**
	United States[Brazil:Rio Grande do Sul:Esteio:2007]	SPLCV-US[BR:RS:Est1:07]
	United States[Brazil:Rio Grande do Sul:Rosario:2007]	SPLCV-US[BR:RS:Ros1:07]
	United States[Brazil:Rio Grande do Sul:Machado Assis1:2007]	SPLCV-US[BR:RS:Mac1:07]
	**United States[Brazil:Sao Paulo:Alfredo Marcondes1:2009]**	**SPLCV-US[BR:SP:AM1:09]**
	**United States[Brazil:Sao Paulo:Alfredo Marcondes2:2009]**	**SPLCV-US[BR:SP:AM2:09]**
	United States[Brazil:Rio Grande do Sul:Porto Alegre1:2007]	SPLCV-US[BR:RS:Poa1:07]
	**United States[Brazil:Sao Paulo:Alfredo Marcondes4:2009]**	**SPLCV-US[BR:SP:AM4:09]**
	**United States[Brazil:Sao Paulo:Alfredo Marcondes3:2009]**	**SPLCV-US[BR:SP:AM3:09]**
	United States[Korea:Haenam1:09]	SPLCV-US[KR:Hae1:09]
	United States[Korea:Yeojoo507:09]	SPLCV-US[KR:Yeo507:09]
	United States[Korea:Yeoju508:09]	SPLCV-US[KR:Yeo508:08]
	United States[Korea:Haenam532:09]	SPLCV-US[KR:Hae532:09]
	United States[Korea:Haenam519:09]	SPLCV-US[KR:Hae519:09]
	United States[Korea:Yeojoo388:09]	SPLCV-US[KR:Yeo388:09]
	United States[Korea:Nonsan445-2:09]	SPLCV-US[KR:Non445-2:09]
	United States[Korea:Chungju263:09]	SPLCV-US[KR:Chu263:09]
	United States[Korea:Haenam618:09]	SPLCV-US[KR:Hae618:09]
	United States[United States:Louisiana:1994]	SPLCV-US[US:Lou:94]
	**United States[Brazil:Rondonia:Ouro Preto do Oeste:2008]**	**SPLCV-US[BR:RO:OPO:08]**
	United States[China:Jiangsu:2008]	SPLCV-US[CN:Jia:08]
	United States[Japan:Kyoto:1998]	SPLCV-US[JR:Kyo:98]
	United States[United States:Mississippi:WS1-4:2007]	SPLCV-US[US:MS:WS1-4:07]
	United States[United States:Mississippi:WS3-8:2007]	SPLCV-US[US:MS:WS3-8:07]
	United States[United States:Mississippi:WS4b-14:2007]	SPLCV-US[US:MS:4b-14:07]
	United States[United States:Mississippi:WS1b-1a:2007]	SPLCV-US[US:MS:1b-1a:07]
	China[China:Yunnan:RL7:2007]	SPLCV-CN[CN:Yn:RL7:07]
	China[China:Yunnan338:2009]	SPLCV-CN[CN:Yn338:09]
	China[China:Yunnan:RL31:2007]	SPLCV-CN[CN:Yn:RL31:07]
	Sao Paulo[Brazil:Sao Paulo:Alvares Machado:2009]	SPLCV-SP[BR:SP:AlvM:09]
	**Sao Paulo[Brazil:Sao Paulo:Presidente Prudente:2009]**	**SPLCV-SP[BR:SP:PP:09]**
	Japan[Japan:Kumamoto:1998]	SPLCJV-JP[JR:Kum:98]
	Japan[Japan:Miyazaki:1996]	SPLCJV-JP[JR:Miy:96]
	South Carolina[United States:South Carolina:646-B11:2006]	SPLCV-SC[US:SC:646-B11:06]
	South Carolina[United States:South Carolina:634-7:2006]	SPLCV-SC[US:SC:634-7:06]
	South Carolina[United States:South Carolina:377-23:2006]	SPLCV-SC[US:SC:377-23:06]
	South Carolina[United States:South Carolina:634-2:2006]	SPLCV-SC[US:SC:634-2:06]
	**Brazil[Brazil:Bahia:Cruz das Almas:2008]**	**SPLCV-BR[BR:BA:CA:08]**
	**Brazil[Brazil:Rondonia:Cacoal:2008]**	**SPLCV-BR[BR:RO:Cac:08]**
	**Brazil[Brazil:Rondonia:Ouro Preto do Oeste:2008]**	**SPLCV-BR[BR:RO:OPO:08]**
	**Brazil[Brazil:Sergipe:Riachao:2008]**	**SPLCV-BR[BR:SE:Ria:08]**
	**Brazil[Brazil:Bahia:Urucuca:2008]**	**SPLCV-BR[BR:BA:Uru:08]**
	Puerto Rico[Puerto Rico:Merremia-N4:2006]	SPLCV-PR[PR:Me-N4:06]
	Puerto Rico[Puerto Rico:80-N2:2006]	SPLCV-PR[PR:80-N2:06]
	**Pernambuco[Brazil:Rondonia:Porto Velho:2008]**	**SPLCV-PE[BR:RO:PV:08]**
	**Pernambuco[Brazil:Rio grande do Sul:Mariana Pimentel1:2009]**	**SPLCV-PE[BR:RS:MP1:09]**
	**Pernambuco[Brazil:Rio Grande do Sul:Mariana Pimentel2:2009]**	**SPLCV-PE[BR:RS:MP2:09]**
	**Pernambuco[Brazil:Rio Grande do Sul:Mariana Pimentel5:2009]**	**SPLCV-PE[BR:RS:MP5:09]**
	**Pernambuco[Brazil:Pernambuco:Camocin de São Félix:2009]**	**SPLCV-PE[BR:PE:CSF1:09]**
	**Pernambuco[Brazil:Pernambuco:Camocin de São Félix:2009]**	**SPLCV-PE[BR:PE:CSF2:09]**
	**Pernambuco[Brazil:Paraiba:Pedras de Fogo:2009]**	**SPLCV-PE[BR:PB:PF:09]**
	**Pernambuco[Brazil:Rio Grande do Sul:Mariana Pimentel4:2009]**	**SPLCV-PE[BR:RS:MP4:09]**
	**Pernambuco[Brazil:Rio Grande do Sul:Mariana Pimentel7:2009]**	**SPLCV-PE[BR:RS:MP7:09]**
	**Pernambuco[Brazil:Rio Grande do Sul:Mariana Pimentel6:2009]**	**SPLCV-PE[BR:RS:MP6:09]**
	**Pernambuco[Brazil:Rio Grande do Sul:Mariana Pimentel3:2009]**	**SPLCV-PE[BR:RS:MP3:09]**
	Fujian[China:Jujian:Fp3:2007]	SPLCV-Fu[CN:Fuj:Fp3:07]
	Fujian[China:Jujian:Fp2:2007]	SPLCV-Fu[CN:Fuj:Fp2:07]
	Fujian[China:Jujian:Fp1:2007]	SPLCV-Fu[CN:Fuj:Fp1:07]
	Spain[Spain:Canary Islands:BG12:2002]	SPLCV-ES[ES:CI:BG12:02]
	Spain[Spain:Canary Islands:BG6:2002]	SPLCV-ES[ES:CI:BG6:02]
	Spain[Spain:Canary Islands:BG13:2002]	SPLCV-ES[ES:CI:BG13:02]
	Italy[Italy:Sicily:2002]	SPLCV-IT[IT:Sic:02]
Sweet potato leaf curl Lanzarote virus	Brazil[Brazil:Rio Grande do Sul:Tavares1:2007]	SPLCLaV-BR[BR:RS:Tav1:07]
	Spain[Spain:Canary Islands:BG27:2002]	SPLCLaV-ES[ES:CI:BG27:02]
	Spain[Spain:Malaga:BG30:2006]	SPLCLaV-ES[ES:Mal:BG30:06]
Sweet potato leaf curl Bengal virus	[india:West Bengal:2008]	SPLCBeV-[IN:Ben:08]
Sweet potato leaf curl Brazil virus	[Brazil:Ceara:Fortaleza1:2008]	SPLCBRV-[BR:CE:For1:08]
Sweet potato leaf curl Canary virus	[Spain:Canary Islands:BG4:2002]	SPLCCaV-[ES:CI:BG4:02]
	[Spain:Canary Islands:BG7:2002]	SPLCCaV-[ES:CI:BG7:02]
	[Spain:Canary Islands:BG21:2002]	SPLCCaV-[ES:CI:BG21:02]
	[Spain:Canary Islands:BG25:2002]	SPLCCaV-[ES:CI:BG25:02]
Sweet potato leaf curl Shanghai virus	[China:Shanghai:2007]	SPLCShV-[CN:Sha:07]
Sweet potato leaf curl Georgia virus	[United States:Georgia:16]	SPLCGoV-[US:Geo:16]
Merremia leaf curl virus	United States[United States:Mississippi:1B-3:2007]	MerLCuV-US[US:MS:1B-3:07]
	Brazil[Brazil:Para:Belem1:2007]	MerLCuV-BR[BR:PA:Bel1:07]
	Puerto Rico[Puerto Rico:N1:2006]	MerLCuV-PR[PR:N1:06]
Ipomoea yellow vein Malaga virus	[Spain:Malaga:IG1:2006]	IYVMaV-[ES:Mal:IG1:06]
Ipomoea yellow vein virus	[Spain:Malaga:IG5:2006]	IYVV-[ES:Mal:IG5:06]
	[Spain:1998]	IYVV-[ES:98]
	[Spain:Malaga:IG3:2006]	IYVV-[ES:Mal:IG3:06]
Sweet potato leaf curl Sao Paulo virus	[Brazil:Sao Paulo:Alvares Machado:2009]	SPLCSPV-[BR:SP:AlvM:09]
Sweet potato mosaic virus	[Brazil:Brasilia1:2007]	SPMV-[BR:BSB1:07]
Sweet potato leaf curl South Carolina virus	[United States:South Carolina:648-B9:2006]	SPLCSCV-[US:SC:648-B9:06]
Sweet potato leaf curl Uganda virus	[Uganda:Kampala:2008]	SPLCUV-[UG:KAMP:08]
Sweet potato leaf curl Spain virus	[Spain:Canary Islands:BG1:2002]	SPLCESV-[ES:CI:BG1:02]
	[Spain:Canary Islands:BG5:2002]	SPLCESV-[ES:CI:BG5:02]
	**[Brazil:Bahia:Utinga:2008]**	**SPLCESV-[BR:BA:Uti:08]**
	[Spain:Malaga:IG2:2006]	SPLCESV-[ES:Mal:IG2:06]
	**[Brazil:Rondonia:Cacoal:2008]**	**SPLCESV-[BR:RO:Cac:08]**
Sweet potato golden vein virus	**Paraiba[Brazil:Bahia:Cruz das Almas:2008]**	**SPGVV-PB[BR:BA:CA:08]**
	Paraiba[Brazil:Paraiba:Souza1:2007]	SPGVV-PB[BR:PB:Sou1:07]
	**Rondonia[Brazil:Rondonia:Cacoal:2008]**	**SPGVV-RO[BR:RO:Cac:08]**
	**Rondonia[Brazil:Rondonia:Porto Velho2:2008]**	**SPGVV-RO[BR:RO:PV2:08]**
	**Rondonia[Brazil:Rondonia:Ouro Preto do Oeste:2008]**	**SPGVV-RO[BR:RO:OPO:08]**
	**Rondonia[Brazil:Bahia:Utinga:2008]**	**SPGVV-RO[BR:BA:Uti:08]**
	**Rondonia[Brazil:Rondonia:Porto Velho 1:2008]**	**SPGVV-RO[BR:SE:PV1:08]**
Sweet potato leaf curl China virus	[China:2005]	SPLCCNV-[CN:05]
	[China:Zhejiang:2010]	SPLCCNV-[CN:Zhe:10]

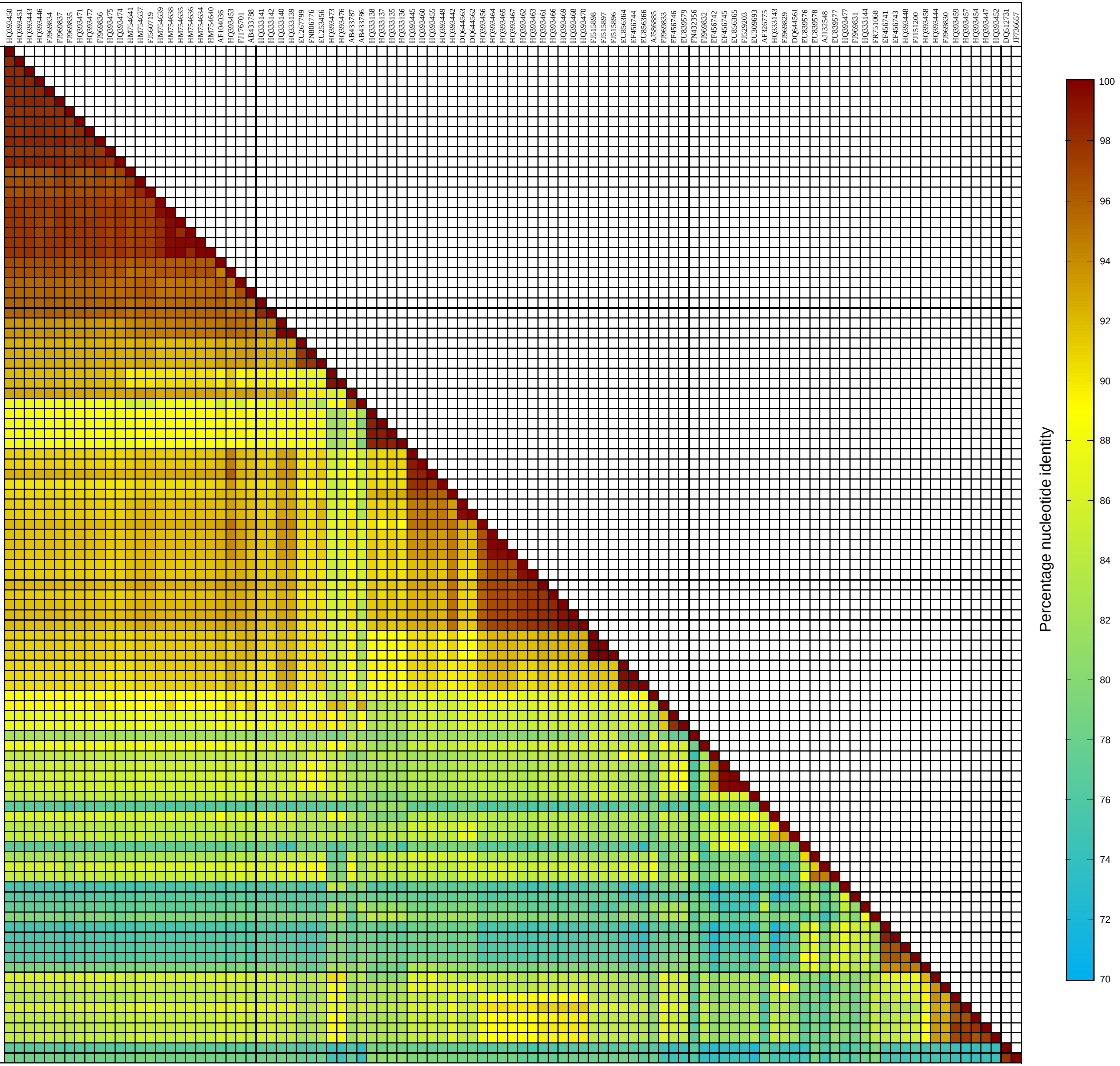

Supplement: Additional file 2 — Sweepovirus information: Complete names and color representations of the pairwise sequence identity percentages (calculated with Clustal V, included in MegAlign-DNASTAR) of the complete genome sequences of sweepovirus isolates reported here and those available in the public sequence databases. The isolates present in this study are shown in bold. [file 1743-422X-9-241-S2.pdf]
